# Supplementary material for: Development of Next-Generation Probiotics by Investigating the Interrelationships between Gastrointestinal Microbiota and Diarrhea in Preruminant Holstein Calves
Source: Animals (Basel). 2022 Mar 10;12(6):695. doi: 10.3390/ani12060695 (PMC8944458; doi:10.3390/ani12060695)
Supplement: Supplementary file 1 [file animals-12-00695-s001.zip › animals-1568968-supplementary.pdf]

Table S1. The information of calf health status

Sample	Age(day)	Starter(Y/N)	Health status[1]	Fecal score
H01	32	Y	H	1
H02	40	Y	H	0
H03	30	Y	H	1
H04	51	Y	H	0
H05	39	Y	H	1
H06	31	Y	H	0
H07	43	Y	H	1
H08	43	Y	H	0
H09	35	Y	H	0
H10	41	Y	H	0
D01	30	Y	D	2
D02	30	Y	D	3
D03	44	Y	D	2
D04	49	Y	D	2
D05	41	Y	D	2
D06	48	Y	D	2
D07	38	Y	D	2
D08	33	Y	D	2
D09	35	Y	D	3
D10	33	Y	D	2

[1] H: Health; D: Diarrhea

Table S2. The relative abundances (%) of dominant taxa in 20 fecal samples at genus and species levels

Taxa	Whole(n=20)		Health(n=10)		Diarrhea(n=10)		P-value	Significance
	Mean	SD	Mean	SD	Mean	SD		
**Genus**								
Faecalibacterium	15.31	6.98	16.72	5.54	13.90	7.80	0.248	-
Prevotella_9	9.45	7.27	12.70	7.67	6.20	5.13	0.075	#
Bacteroides	9.09	5.80	8.60	6.36	9.58	5.20	0.631	-
Prevotella_2	6.32	5.12	6.83	6.12	5.82	3.96	0.684	-
Ruminococcaceae_UCG_014	6.06	4.15	8.81	3.32	3.31	2.77	0.002	**
Megamonas	5.74	5.32	6.13	4.31	5.35	6.06	0.342	-
Alloprevotella	5.07	5.28	2.71	3.74	7.43	5.41	0.035	*
Blautia	3.49	1.97	3.49	2.46	3.49	1.39	0.529	-
Parabacteroides	2.82	2.36	2.69	3.05	2.95	1.46	0.280	-
Phascolarctobacterium	2.81	2.00	2.83	2.44	2.79	1.51	0.631	-
Bifidobacterium	2.45	2.11	3.56	2.22	1.33	1.23	0.015	*
Rikenellaceae_RC9_gut_group	1.58	2.64	1.62	3.28	1.53	1.89	0.723	-
Holdemanella	1.47	2.24	1.96	2.84	0.99	1.35	0.868	-
Sutterella	1.47	1.62	1.88	2.00	1.05	1.01	0.315	-
Megasphaera	1.43	2.32	1.88	2.85	0.98	1.59	0.481	-
Collinsella	1.31	1.38	1.18	0.51	1.45	1.83	0.481	-
Anaerovibrio	0.93	1.63	0.75	0.97	1.11	2.03	0.896	-
Subdoligranulum	0.86	1.05	0.45	0.56	1.27	1.22	0.043	*

Succinivibrio	0.83	2.42	0.29	0.29	1.37	3.23	0.752	-
Tyzzerella_4	0.69	0.96	0.59	0.75	0.79	1.17	0.796	-
[Ruminococcus]_gnavus_group	0.64	0.65	0.77	0.42	0.51	0.82	0.092	#
Ruminococcaceae_UCG_005	0.53	1.16	0.07	0.19	1.00	1.53	0.003	**
Ruminococcus_2	0.53	0.63	0.28	0.38	0.77	0.75	0.023	*
Fournierella	0.51	0.73	0.39	0.40	0.62	0.97	0.853	-
Lachnoclostridium	0.47	0.50	0.26	0.20	0.67	0.62	0.046	*
Prevotella_7	0.46	0.74	0.37	0.63	0.56	0.85	0.105	-
Odoribacter	0.46	0.75	0.14	0.35	0.77	0.93	0.036	*
Corynebacterium_1	0.44	1.92	0.00	0.00	0.88	2.72	0.225	-
Sharpea	0.42	0.60	0.55	0.78	0.29	0.34	0.895	-
Acidaminococcus	0.42	0.65	0.39	0.39	0.44	0.87	0.217	-
Ruminiclostridium_9	0.37	0.22	0.22	0.13	0.53	0.19	<0.001	***
Pseudoflavonifractor	0.37	0.34	0.51	0.40	0.23	0.22	0.030	*
Lachnospiraceae_NK4A136_group	0.36	1.52	0.01	0.03	0.71	2.14	0.004	**
Escherichia_Shigella	0.34	0.53	0.22	0.45	0.47	0.59	0.063	#
Alistipes	0.33	0.42	0.23	0.44	0.44	0.40	0.211	-
Tyzzerella	0.31	0.34	0.24	0.22	0.39	0.43	0.425	-
Olsenella	0.31	0.35	0.20	0.22	0.42	0.43	0.218	-
Fusobacterium	0.31	0.82	0.03	0.04	0.60	1.12	0.332	-
Erysipelatoclostridium	0.31	0.33	0.22	0.25	0.39	0.38	0.481	-
Succiniclasticum	0.30	0.72	0.24	0.37	0.35	0.97	0.670	-

Helicobacter	0.29	1.06	0.08	0.16	0.50	1.50	0.166	-
Erysipelotrichaceae_UCG_003	0.28	0.56	0.42	0.73	0.15	0.28	0.740	-
Dialister	0.28	0.73	0.13	0.39	0.43	0.97	0.844	-
Oscillospira	0.23	0.23	0.22	0.25	0.25	0.21	0.424	-
Prevotellaceae_NK3B31_group	0.22	0.57	0.01	0.02	0.44	0.76	0.054	#
[Ruminococcus]_torques_group	0.22	0.21	0.31	0.25	0.13	0.12	0.063	#
[Ruminococcus]_gauvreauii_group	0.21	0.30	0.10	0.15	0.33	0.37	0.050	*
Desulfovibrio	0.21	0.55	0.10	0.10	0.33	0.77	0.925	-
Pelistega	0.20	0.78	0.04	0.05	0.37	1.11	0.404	-
Negativibacillus	0.20	0.14	0.16	0.13	0.24	0.15	0.403	-
Lactobacillus	0.20	0.49	0.06	0.07	0.34	0.68	0.986	-
Akkermansia	0.20	0.67	0.07	0.14	0.33	0.94	0.473	-
Faecalicoccus	0.19	0.23	0.18	0.17	0.20	0.29	0.353	-
Barnesiella	0.17	0.32	0.12	0.25	0.22	0.38	0.278	-
Ruminiclostridium_5	0.16	0.22	0.15	0.23	0.17	0.22	0.739	-
Dorea	0.16	0.12	0.19	0.12	0.13	0.12	0.315	-
Prevotella_1	0.15	0.35	0.16	0.43	0.15	0.27	0.410	-
Butyricimonas	0.14	0.21	0.08	0.14	0.20	0.25	0.590	-
Ruminococcus_1	0.12	0.53	0.00	0.00	0.24	0.74	0.211	-
**Species**								
Bifidobacterium_longum_subsp_longum	1.89	1.96	2.91	2.00	0.86	1.34	0.009	**
Megasphaera_elsdenii	1.28	2.10	1.67	2.57	0.89	1.54	0.618	-
Bacteroides_plebeius_DSM_17135	1.04	1.60	1.77	2.01	0.31	0.41	0.279	-

Clostridium_sp_AUH_JLC235	0.84	0.65	0.83	0.53	0.86	0.78	0.912	-
Bacteroides_fragilis	0.48	1.25	0.21	0.51	0.75	1.70	0.645	-
Bacteroides_thetaiotaomicron	0.48	0.69	0.45	0.90	0.51	0.43	0.063	-
bacterium_ic1277	0.44	0.62	0.17	0.31	0.71	0.74	0.018	*
Corynebacterium_sp	0.41	1.80	0.00	0.00	0.82	2.54	0.141	-
Bacteroidaceae_bacterium_DJF_B220	0.23	1.02	0.00	0.00	0.46	1.45	0.999	-
Parabacteroides_distasonis	0.21	0.20	0.21	0.22	0.20	0.18	0.971	-
unidentified_rumen_bacterium_JW32	0.14	0.51	0.23	0.71	0.05	0.12	0.582	-
Bifidobacterium_animalis	0.07	0.27	0.02	0.05	0.13	0.37	0.590	-
Prevotella_sp_DJF_CP65	0.06	0.14	0.01	0.02	0.11	0.18	0.696	-
Lactobacillus_mucosae	0.04	0.15	0.01	0.01	0.07	0.21	0.163	-
butyrate_producing_bacterium_L2_10	0.04	0.08	0.01	0.02	0.07	0.10	0.106	-
Lactobacillus_murinus	0.04	0.07	0.02	0.02	0.06	0.10	0.558	-
Clostridium_sp_K4410MGS_306	0.04	0.07	0.01	0.02	0.07	0.09	0.085	-
Lactobacillus_reuteri	0.03	0.07	0.01	0.01	0.05	0.09	0.598	-
Gallibacterium_genomosp_3	0.03	0.08	0.01	0.02	0.04	0.11	0.894	-
Corynebacterium_amycolatum	0.03	0.11	0.00	0.00	0.05	0.15	0.195	-

Data were expressed as mean ± SD. Mann-Whitney test was applied to evaluate the significant difference between the healthy and diarrheic groups.

Significant difference: * $P < 0.05$; ** $P < 0.01$; *** $P < 0.001$.
